# The application of artificial intelligence in diabetic retinopathy screening: a Saudi Arabian perspective

**DOI:** 10.3389/fmed.2023.1303300

**Published:** 2023-11-22

**Authors:** Abdulaziz A. Barakat, Omar Mobarak, Haroon Ahmed Javaid, Mhd Rasheed Awad, Karam Hamweyah, Abderrahman Ouban, Selwa A. F. Al-Hazzaa

**Affiliations:** ^1^College of Medicine, Alfaisal University, Riyadh, Saudi Arabia; ^2^King Abdulaziz City for Science and Technology (KACST), Riyadh, Saudi Arabia

**Keywords:** diabetic retinopathy, artificial intelligence, screening, Saudi Arabia, ophthalmology, COVID-19, pandemic

## Abstract

**Introduction:**

Diabetic retinopathy (DR) is the leading cause of preventable blindness in Saudi Arabia. With a prevalence of up to 40% of patients with diabetes, DR constitutes a significant public health burden on the country. Saudi Arabia has not yet established a national screening program for DR. Mounting evidence shows that Artificial intelligence (AI)-based DR screening programs are slowly becoming superior to traditional screening, with the COVID-19 pandemic accelerating research into this topic as well as changing the outlook of the public toward it. The main objective of this study is to evaluate the perception and acceptance of AI in DR screening among eye care professionals in Saudi Arabia.

**Methods:**

A cross-sectional study using a self-administered online-based questionnaire was distributed by email through the registry of the Saudi Commission For Health Specialties (SCFHS). 309 ophthalmologists and physicians involved in diabetic eye care in Saudi Arabia participated in the study. Data analysis was done by SPSS, and a value of *p* < 0.05 was considered significant for statistical purposes.

**Results:**

54% of participants rated their level of AI knowledge as above average and 63% believed that AI and telemedicine are interchangeable. 66% believed that AI would decrease the workforce of physicians. 79% expected clinical efficiency to increase with AI. Around 50% of participants expected AI to be implemented in the next 5 years.

**Discussion:**

Most participants reported good knowledge about AI. Physicians with more clinical experience and those who used e-health apps in clinical practice regarded their AI knowledge as higher than their peers. Perceived knowledge was strongly related to acceptance of the benefits of AI-based DR screening. In general, there was a positive attitude toward AI-based DR screening. However, concerns related to the labor market and data confidentiality were evident. There should be further education and awareness about the topic.

## 1 Introduction

Diabetic Retinopathy (DR), the most common microvascular complication of Diabetes Mellitus (DM), is a leading cause of preventable blindness in adults in the working-age group (1). A recent study concluded that globally, around 22.3% of patients with diabetes were estimated to develop DR in 2021 (2). In Saudi Arabia, the prevalence of DR is generally higher, ranging from around 20 to 45% (3). Screening for DR is required to stage the disease and make timely referrals, which typically involves visual acuity and retinal examination (ophthalmoscopy or fundus photography), with optional supplementary optical coherence tomography (OCT) (4). The American Academy of Ophthalmology recommends annual DR screening from 5 years after the onset of disease for type 1 diabetes and from the time of diagnosis for type 2 diabetes (5).

The first recorded attempts to establish a screening system for DR were in Iceland in 1980, often considered the pioneers of DR screening, with 90% of all type 1 diabetics in the country undergoing annual fundoscopy and eye exams with subsequent treatment of any noted proliferative DR and macular edema. It has been proposed by Kristinsson et al. that the significantly lower rates of legal blindness in Iceland compared to other Nordic countries were attributable, at least in part, to the aggressive and early examination of DR (6). Ever since then, multiple screening programs have been developed worldwide to achieve similar success to Iceland’s. The United Kingdom (UK) accounts for one of the largest and most successful public health initiatives directed toward DR screening, with a nationwide program starting in the year 2003 aiming to screen all DM patients above the age of 12. The screening was done by 45-degree digital fundus cameras with pupillary dilation. The national uptake of this program was upwards of 80% (7), covering 2.59 million people as of 2016 (8). When we start to look locally at Saudi Arabia, we see that despite the rising prevalence of DM, there has been a paucity of studies to properly assess the presence of DR, except for one report from a tertiary care center in Riyadh (9). Although an estimated 17.7% of the population had DM as of 2021, screening prevalence was reported from 15.2 to 25% of cases in the primary care setting. However, this study was limited by its small sample size (10). Consequently, patients tend to present with more severe forms of DR whose progression could have been either slowed or halted had screening been undergone earlier. Screening prevalence declines even further when we consider common barriers to screening, such as poor socioeconomic status, transportation difficulty, or mental health issues such as depression (11). Naturally, the consideration of AI-based screening models for DR is one potential solution to solve the poor screening uptake we see locally.

AI-based systems have been studied in multiple medical fields, including radiology, dermatology, and ophthalmology with varying success, and with the potential to be used in many other domains. In a study by Shaffer et al., a sample of 144,231 screening mammograms from 85,580 women was used to train a number of AI algorithms and compare their performance to human graders. Although no one algorithm was found to be superior to human graders, a composite of all the trained algorithms was able to reach a higher sensitivity compared to single-radiologist assessment (12). The potential scope of artificial intelligence has even expanded to include the field of cardiology. In a systematic review by Garavand et al. 54 studies were reviewed discussing machine learning algorithms in the diagnosis of coronary artery disease. The authors predicted that machine learning and deep-learning based diagnostic models would soon grow to be essential tools in the diagnosis of coronary artery disease (13). Another study documented sensitivities as high as 92%, and an accuracy of prediction of 85% in the diagnosis of coronary artery disease (14). The COVID-19 pandemic, owing to its rapid acceleration of technological development worldwide, has only served to fuel further speculation and research regarding the utility of artificial intelligence in streamlining clinical workflow. In a systematic review of 60 studies evaluating the performance of deep-learning models in the diagnosis of COVID-19 via plain radiographs, AI-based diagnostic algorithms were found to have a sensitivity of over 96%, and a specificity of 92% (15). Wang et al. performed a meta-analysis on 78 studies tackling AI performance in COVID-19 diagnosis, yielding similarly high sensitivities and specificities, at 75.00–99.44% and 71.80–100.00%, respectively (16).

The efficacy of AI lies primarily in its ability of risk stratification, as well as in its potential for deep learning. Deep learning allows the analysis of complex images accurately, which was once an operator-dependent task, and spares specialists’ time for more complex procedures. It is estimated that AI-based systems will overtake human intelligence in efficiency soon, with current models already matching expert opinion in accuracy (17). The most primitive attempts to integrate the use of AI into the field of ophthalmology began in 1973, involving the use of computer-aided detection (CAD) to detect qualitative features like contour lines in scans of patients’ retinas. Ever since, AI programs continued to evolve, becoming much more sophisticated and providing substantial quantitative data valuable in assessing patient risk.

The adaptation toward AI naturally extended to the screening of DR. Typically, the screening process for DR involves the capture of retinal photographs, which are then independently graded by trained technicians. This creates a natural labor sink where a large amount of resources need to be poured in order to effectively screen for the disease at the population level (18). In addition, despite the necessity for high diagnostic specificity, approximately 5% of referrable DR cases were missed by experts (19). AI implementation allows a practical solution for these problems, limiting the amount of human input necessary to accurately diagnose DR. One of the earliest trials published in the development of AI programs in DR screening was by Abramoff et al. in 2008, demonstrating an 84% sensitivity and 64% specificity in diagnosing DR. These programs are often termed “ARIAS” (Automated Retinal Image Analysis System) (7), and comprise multiple different pieces of software including IDx-DR, RetmarkerDR, EyeArt, and a neural network developed by Google, and were the steppingstone to the development of automated screening systems. These systems primarily assess DR via the following (20, 21):

- A dataset (Often a public one such as Messidor or Messidor-2) is fed to the program, often with annotated lesions to “train” the neural network.
- The program learns patterns associated with the presence or absence of disease based on complex mathematical algorithms.
- The results are usually compared against a reference dataset.
- The program automatically corrects for errors that may influence its results negatively.
- A unique output is generated by the program in the form of specific lesion detection^1^ (Macular edema, hemorrhages, exudates, etc.…), sorting images based on disease state (see Footnote 1), or other outputs, and generating a referral recommendation.

With improvements in deep learning, more complex problems can be solved, improving the specificity of available systems. A report by Abramoff et al. reported the diagnostic sensitivity and specificity following the integration of deep learning into their systems (Based on IDx-DR 2.1) were 96.8 and 87%, respectively (22). Similarly high diagnostic accuracies have been achieved through the implementation of deep learning in other screening programs, with most of these results being externally validated against human graders (23–25).

Multiple studies have aimed to directly compare AI-based DR screening models to human graders in realistic and practical scenarios to assess their suitability for real-life applications. In a trial by Soto-Pedre et al. in Spain comparing the results of an automated screening system (iGrading, Medalytix Ltd., Manchester-UK) to the results of human graders following the national screening program, automated screening was almost as sensitive (94.5%) and less specific (68.8%) as human graders (18). The relatively low specificity was attributed to the higher prevalence of macular degeneration and arteriovenous crossing in the study sample. It was concluded that AI implementation would reduce ophthalmologist workload by 44%. Another study published in China further solidified the reliability of AI in DR screening, suggesting an overall accuracy of 75% using the CARE system in Chinese community health centers (26). One of the most extensive real-life studies performed on over 100,00 consecutive patient visits suggested a sensitivity of 91.3% and specificity of 91.1% using the EyeArt 2.0 system (27) as a follow-up to an earlier UK study analyzing the effectiveness of EyeArt 1.0 on 20,000 patient visits (28).

Of note, a recent prospective trial validating the effectiveness of AI in real-life clinical scenarios was published in 2022, spanning 7,651 patients and evaluated the feasibility of implementing an AI DR screening program (based on Google’s neural networks) in Thailand, widely known to be a middle-income country, filling a gap in the literature regarding the utility of these systems in less economically affluent countries which are generally disproportionately affected by diabetic retinopathy. The results of this study showed improved sensitivity and similar specificity to Thai retina specialists, with a practical yet slightly more challenging applicability in Thailand considering the current socio-economic standing (24).

Although AI systems have demonstrated low diagnostic accuracy outside of the lab in some studies (29), accuracy is demonstrably at least as efficient as human graders in real-world performance, and applicable with preserved robustness in the face of variations in clinical characteristics of different patients (27). A meta-analysis of most available studies comparing human graders to automated DR screening programs found no significant difference in heterogeneous diagnostic accuracy (28).

The challenge of using AI in healthcare is to address the concerns, preconceived notions, and unmet needs of various stakeholders, such as patients, clinicians, governmental or ethical entities, software developers, etc. Therefore, introducing Al to the healthcare system without addressing these concerns will lead to an untenable system. To our knowledge, there is a lack of studies specifically addressing the perceptions of AI-based DR screening. As the scope of AI is expanding, we have started seeing a shift in the focus of researchers toward studying the acceptance of these technologies among both the general population and clinicians alike. At the patient level, AI-based screening is generally well-accepted among patients of various backgrounds and ethnicities. Studies from Australia, New Zealand, and Asia reinforce this notion, albeit with some gaps in knowledge that may make patients somewhat hesitant to consider the reliability of AI-based technologies, possibly due to poor awareness (30, 31). Despite these concerns, recent evidence suggests that using AI-based screening can improve patient compliance with screening recommendations (32, 33).

Studies specifically aiming to look at clinician perceptions of AI-based screening, on the other hand, are severely lacking. This poses a significant challenge to the development of nationwide AI-based screening protocols, as considering these perceptions and recognizing knowledge gaps will be necessary for regulatory bodies, healthcare systems, educators, AI developers, and professional entities. Our descriptive study aims to remedy this gap, creating a more solid bedrock to consider the implementation of this technology.

## 2 Materials and methods

### 2.1 Setting and design

A cross-sectional study was conducted from September 2020 to March 2022 among the ophthalmologists, family physicians, endocrinologists and general physicians involved in managing patients with diabetes across Saudi Arabia. The questionnaire was generated via Google Forms (34). The cross-sectional study comprised of 23 multiple-choice items related to demographic characteristics, profession, years of experience, perceived knowledge about AI in diabetic retinopathy, and the experts’ assessment of AI’s potential benefits, disadvantages, supporting infrastructure, and readiness for adoption in the real-world clinical setting. The use of e-health apps and their correlation with the aforementioned variables was also investigated. To clarify, “e-health app” is a broad umbrella of digital applications that can range from allowing a platform for users to interface with their healthcare services, such as viewing patient medical records, chatting with healthcare providers, and booking primary care clinic visits, to tracking health statistics.

Informed consent was included in the introduction to the questionnaire, briefing participants about the research purpose and ethical considerations (Appendix 1). Participation was voluntary by answering the questionnaire and submitting it. The data collected was confidential and only the primary investigator had access to the data file. In addition, the data was not used for purposes other than the study. The study was approved by Alfaisal University’s Institutional Review Board (IRB) with the identification number IRB-20063.

Face validity of the questionnaire was established by an expert in DR in Saudi Arabia. A pilot study was conducted to test the questionnaire by distributing it to a sample of ophthalmologists (*n* = 11). Data collection and cleaning were done by Excel spreadsheet. Common themes for the questions measuring the same concept were identified by principal components analysis (PCA) (Supplementary Figure S1). The reliability of the questionnaire results and internal consistency of the questions loaded into the same factors were tested by Cronbach’s Alpha. Overall Cronbach’s Alpha for the questionnaire was 0.887 which indicated excellent reliability in the responses (Appendix 2), and the values of Cronbach’s Alpha for internal consistency of the factor themes (Appendix 3) were all above 0.6. Questions 12, 13,14, and 21 were loaded onto the same factor (which represented the advantages of AI application to clinical practice), and Cronbach’s Alpha was 0.816. In addition, questions 15, 16, and 17 were grouped into the same factor (which represented implementation and education of AI screening in the next 5 years) and Cronbach’s Alpha was 0.918. Questions 18, 19, and 20 represented the advantages of AI screening, and Cronbach’s Alpha was 0.773. Questions 22 and 23 represented the safety of AI in COVID-19 pandemic, and Cronbach’s Alpha was 0.790. Questions 10 and 11 represented the effect of AI on the labor market and Cronbach’s Alpha was 0.662. Supplementary Figure S2 summarizes the research pipeline.

### 2.2 Data collection

The target population of this study included all ophthalmologists and physicians involved in the care of diabetic patients in Saudi Arabia, such as general practitioners, family physicians, and endocrinologists. A sample size of 270 ophthalmologists was considered based on a 95% confidence level, a 5% margin of error, and assuming a total of 900 registered members of the Saudi Ophthalmological Society with a 50% response distribution (35). Sample size calculation was performed by using an online Raosoft sample size calculator (36). The online questionnaire was distributed to physicians by email through the registry of the Saudi Commission for Health Specialties (SCFHS) starting in September 2020. Reminder emails were sent until we reached the desired sample size in March 2022.

### 2.3 Data analysis and management

Descriptive statistics of frequency distribution and percentages were calculated for the demographic variables and AI questionnaire items. A normality check was performed on the data. Categorical variables were analyzed by using the Chi-square test. The dataset was assumed to be normally distributed. This was based on the calculation of our sample’s skewness and kurtosis values for each question theme, which were found to be between (−0.390 ≤ γ ≤ −0.278) and (−0.209 ≤ κ ≤ 0.346) respectively, as is outlined (Appendix 4). An asymmetry and kurtosis value between-2 and + 2 were considered as significant at predicting a normal distribution of a given sample. A mean perception score was calculated and compared between demographic variables by applying the independent t-test (current position, practicing in Saudi Arabia, gender, use of e-health) and a one-way ANOVA test was performed. Post-hoc testing was performed for clinical experience in eye care services utilizing the Tukey method. A value of *p* < 0.05 was considered significant for all statistical purposes. Microsoft Excel 365 software was used to collect the data (37). All data were analyzed using SPSS (38).

## 3 Results

The study included 309 respondents who were from Saudi Arabia. These included 267 (86%) ophthalmologists and 42 (14%) others. The majority were males (67%). More than half (53%) had 5 to 20 years of clinical experience in eye care services, while 35% had more than 20 years of experience (Table 1).

**Table 1:** Demographic characteristics of study participants (*N* = 309).

		*n*	%
Current profession	Ophthalmologist	267	86%
	Other	42	14%
Clinical experience in eye care services	<5 years	38	12%
	5–20 years	164	53%
	>20 years	107	35%
Gender	Male	206	67%
	Female	103	33%

Knowledge of AI varied significantly when stratified against years of experience, as shown in Table 2. When asked to rate their understanding of AI, a significantly lower percentage (34%) of the <5 years of experience group rated themselves as above average/excellent as compared to those with 5–20 years (55%) and > 20 years (57%) of experience (*p* = 0.014). 72% of those in the group with experience >20 years reported that AI and telemedicine can be used interchangeably compared to 60% in the 5–20 years and 47% in the group with <5 years of experience (*p* = 0.017). There was a significantly lower percentage in the <5 years of experience group (13%) who agreed that AI would complement their organization’s diabetic clinical eye practice as compared to 48% in the 5–20 years and 39% in the >20 years of experience group (*p* = 0.001).

**Table 2:** Perceived AI knowledge and clinical application by clinical experience.

		Clinical experience in eye care services						Value of *p*
		<5 years (*n* = 38)		5–20 years (*n* = 164)		>20 years (*n* = 107)		
		*n*	%	*n*	%	*n*	%	
Have e-health apps increased the efficiency of your clinical practice?	Disagree/Neutral	1	4%	25	22%	8	12%	**0.04** ^*^
	Agree	24	96%	87	78%	59	88%	
Using e-health apps promotes my understanding and acceptance of utilizing AI in healthcare	Disagree/Neutral	2	8%	27	24%	10	15%	0.097
	Agree	23	92%	85	76%	58	85%	
How would you rate your understanding of AI?	Very poor/Below average	4	11%	24	15%	7	7%	**0.014** ^*^
	Average	21	55%	49	30%	39	36%	
	Above average/Excellent	13	34%	91	55%	61	57%	
AI and telemedicine can be used interchangeably	Disagree/Neutral	20	53%	65	40%	30	28%	**0.017** ^*^
	Agree	18	47%	99	60%	77	72%	
AI will complement my organization’s diabetic clinical eye practice	Disagree/Neutral	6	9%	47	73%	11	17%	**0.001** ^*^
	Agree	32	13%	117	48%	96	39%	

*Based on 205 respondents who answered yes to using e-health applications. Values in bold denote statistical significance, which is represented by a *p*-value < 0.05.

There were significant differences in AI knowledge and clinical practice between those who used e-health apps in their clinical practice compared to those who did not (Table 3). A greater proportion of those who used the e-health apps (83%) agreed that the apps increased the efficiency of clinical practice compared to 68% of those who did not use the apps (*p* = 0.048). Similarly, there was a higher agreement for the statement that the e-health apps increase the acceptance of utilizing AI in healthcare in those who used the apps (81%) compared to 69% in those who did not (*p* = 0.023). A greater proportion of e-health app users (61%) rated their understanding of AI as above average/excellent as compared to 38% of non-users (*p* < 0.001). There were also significant differences for three statements among respondents who were using e-health apps before and during the COVID-19 pandemic compared to those who were not using them (Table 4). 78% of users agreed that AI would be a competitor to diabetic clinical practitioners compared to non-users (*p* = 0.004). 82% of the users agreed that AI would spare specialists efforts spent in triaging compared to 66% of non-users (*p* = 0.002). Similarly, 82% of the users agreed that AI would increase the specialists’ time to utilize their surgical skills better (*p* < 0.001).

**Table 3:** Effect of using e-health applications on AI knowledge and clinical practice.

		Using e-health apps in your clinical practice prior and during the COVID-19 pandemic?				Value of *p*
		No		Yes		
		*n*	%	*n*	%	
Have e-health apps increased the efficiency of your clinical practice?	Disagree/Neutral	9	32%	34	17%	**0.048**
	Agree	19	68%	170	83%	
Using e-health apps promotes my understanding and acceptance of utilizing AI in healthcare	Disagree/Neutral	28	31%	39	19%	**0.023**
	Agree	62	69%	166	81%	
How would you rate your understanding of AI?	Very poor/Below average	19	18%	16	8%	**<0.001**
	Average	46	44%	63	31%	
	Above average/Excellent	39	38%	126	61%	
AI and telemedicine can be used interchangeably	Disagree/Neutral	45	43%	70	34%	0.12
	Agree	59	57%	135	66%	
AI will complement my organization’s diabetic clinical eye practice	Disagree/Neutral	24	23%	40	20%	0.47
	Agree	80	77%	165	80%	

Values in bold denote statistical significance, which is represented by a *p*-value < 0.05.

**Table 4:** Effect of using e-health apps on the perception of participants on the potential impact of AI application in diabetic retinopathy screening.

		Using e-health apps in your clinical practice prior and during the COVID-19 pandemic?				Value of *p*
		No (*n* = 104)		Yes (*n* = 205)		
		*n*	%	*n*	%	
AI will be a competitor to diabetic clinical practitioners	Agree/Neutral	65	63%	160	78%	**0.004**
	Disagree	39	38%	45	22%	
AI will decrease the number of the workforce needed for diabetic clinical eye care	Agree/Neutral	90	87%	167	81%	0.26
	Disagree	14	13%	38	19%	
AI will complement my organization’s diabetic clinical eye practice	Disagree/Neutral	24	23%	40	20%	0.47
	Agree	80	77%	165	80%	
AI will spare specialists’ efforts spent in triaging DR to better utilize time for more surgical procedures	Disagree/Neutral	35	34%	37	18%	**0.002**
	Agree	69	66%	168	82%	
AI will increase the encounters for specialists to better utilize their surgical skills efficiently	Disagree/Neutral	38	37%	36	18%	**<0.001**
	Agree	66	63%	169	82%	
My organization is likely to invest in AI in the clinical practice for DR in the next 5 years	Disagree/Neutral	59	57%	102	50%	0.25
	Agree	45	43%	103	50%	
My organization is likely to train healthcare workers in the use of AI in the next 5 years	Disagree/Neutral	55	53%	86	42%	**0.068**
	Agree	49	47%	119	58%	
My organization is likely to educate the public regarding the use of AI in Ophthalmology in the next 5 years	Disagree/Neutral	54	52%	88	43%	0.13
	Agree	50	48%	117	57%	
AI can maintain the confidentiality of the patients’ data based on e-health data security & privacy governance protocols	Disagree/Neutral	29	28%	48	23%	0.39
	Agree	75	72%	157	77%	
AI will increase the accessibility of DR screening and follow-up at the patient’s own convenience	Disagree/Neutral	18	17%	21	10%	0.08
	Agree	86	83%	184	90%	
AI will increase detection of early stages DR and decrease the progression of advanced stages DR	Disagree/Neutral	19	18%	27	13%	0.23
	Agree	85	82%	178	87%	
AI will accelerate the management for patients requiring urgent intervention	Disagree/Neutral	14	13%	36	18%	0.36
	Agree	90	87%	169	82%	
AI will offer diabetic patients a safer environment for examination during and post COVID-19 pandemic	Disagree/Neutral	15	14%	30	15%	0.96
	Agree	89	86%	175	85%	
AI will further support non-essential contact between eye care providers and patients during and post COVID-19 pandemic	Disagree/Neutral	18	17%	25	12%	0.22
	Agree	86	83%	180	88%	

Values in bold denote statistical significance, which is represented by a *p*-value < 0.05.

The perception of the participants on the impact of AI application in diabetic retinopathy screening was compared to their current profession, years of clinical experience in eye care, and use of e-health apps in clinical practice (Tables 4–6). With regards to the current profession, a greater proportion of respondents who were not ophthalmologists (others group) agreed with the two statements that their organization would be likely to train healthcare workers, and also that the organization would likely educate the public for the use of AI in the next five years (69% vs. 52% for both statements) as compared to the ophthalmologists (*p* = 0.04) as shown in Table 5. While stratifying for clinical experience, the one response demonstrating the most statistical significance was the one pertaining to the statement “AI would complement the organization’s diabetic clinical eye practice (Table 6). The majority (90%) of respondents who had >20 years of experience agreed with the above statement as compared to 84% of those with <5 years and 71% for those with 5–20 years of experience (*p* = 0.001), suggesting that more experienced clinicians tended to be more confident in AI’s ability to benefit patient care.

**Table 5:** Perception of participants on the impact of AI application in diabetic retinopathy screening by profession.

		Current profession				Value of *p*
		Ophthalmologist (*n* = 267)		Other (*n* = 42)		
		*n*	%	*n*	%	
AI will be a competitor to diabetic clinical practitioners	Agree/Neutral	191	72%	34	81%	0.20
	Disagree	76	28%	8	19%	
AI will decrease the number of the workforce needed for diabetic clinical eye care	Agree/Neutral	220	82%	37	88%	0.36
	Disagree	47	18%	5	12%	
AI will complement my organization’s diabetic clinical eye practice	Disagree/Neutral	59	22%	5	12%	0.13
	Agree	208	78%	37	88%	
AI will spare specialists’ efforts spent in triaging DR to better utilize time for more surgical procedures	Disagree/Neutral	65	24%	7	17%	0.27
	Agree	202	76%	35	83%	
AI will increase the encounters for specialists to better utilize their surgical skills efficiently	Disagree/Neutral	64	24%	10	24%	0.98
	Agree	203	76%	32	76%	
My organization is likely to invest in AI in the clinical practice for DR in the next 5 years	Disagree/Neutral	145	54%	16	38%	**0.051**
	Agree	122	46%	26	62%	
My organization is likely to train healthcare workers in the use of AI in the next 5 years	Disagree/Neutral	128	48%	13	31%	**0.04**
	Agree	139	52%	29	69%	
My organization is likely to educate the public regarding the use of AI in Ophthalmology in the next 5 years	Disagree/Neutral	129	48%	13	31%	**0.04**
	Agree	138	52%	29	69%	
AI can maintain the confidentiality of the patients’ data based on e-health data security & privacy governance protocols	Disagree/Neutral	68	25%	9	21%	0.57
	Agree	199	75%	33	79%	
AI will increase the accessibility of DR screening and follow-up at the patient’s own convenience	Disagree/Neutral	32	12%	7	17%	0.40
	Agree	235	88%	35	83%	
AI will increase detection of early stages DR and decrease the progression of advanced stages DR	Disagree/Neutral	40	15%	6	14%	0.91
	Agree	227	85%	36	86%	
AI will accelerate the management for patients requiring urgent intervention	Disagree/Neutral	40	15%	10	24%	0.15
	Agree	227	85%	32	76%	
AI will offer diabetic patients a safer environment for examination during and post COVID-19 pandemic	Disagree/Neutral	37	14%	8	19%	0.38
	Agree	230	86%	34	81%	
AI will further support non-essential contact between eye care providers and patients during and post COVID-19 pandemic	Disagree/Neutral	34	13%	9	21%	0.13
	Agree	191	72%	34	81%	

Values in bold denote statistical significance, which is represented by a *p*-value < 0.05.

**Table 6:** Participants’ perception of the impact of AI application in diabetic retinopathy screening by clinical experience.

		Clinical experience in eye care services						Value of *p*
		<5 years (*n* = 38)		5–20 years (*n* = 164)		>20 years (*n* = 107)		
		*n*	%	*n*	%	*n*	%	
AI will be a competitor to diabetic clinical practitioners	Agree/Neutral	28	74%	122	74%	75	70%	0.73
	Disagree	10	26%	42	26%	32	30%	
AI will decrease the number of the workforce needed for diabetic clinical eye care	Agree/Neutral	31	82%	137	84%	89	83%	0.96
	Disagree	7	18%	27	16%	18	17%	
AI will complement my organization’s diabetic clinical eye practice	Disagree/Neutral	6	16%	47	29%	11	10%	**0.001**
	Agree	32	84%	117	71%	96	90%	
AI will spare specialists’ efforts spent in triaging DR to better utilize time for more surgical procedures	Disagree/Neutral	6	16%	41	25%	25	23%	0.48
	Agree	32	84%	123	75%	82	77%	
AI will increase the encounters for specialists to better utilize their surgical skills efficiently	Disagree/Neutral	13	34%	38	23%	23	21%	0.27
	Agree	25	66%	126	77%	84	79%	
My organization is likely to invest in AI in the clinical practice for DR in the next 5 years	Disagree/Neutral	14	37%	92	56%	55	51%	**0.099**
	Agree	24	63%	72	44%	52	49%	
My organization is likely to train healthcare workers in the use of AI in the next 5 years	Disagree/Neutral	18	47%	78	48%	45	42%	0.66
	Agree	20	53%	86	52%	62	58%	
My organization is likely to educate the public regarding the use of AI in Ophthalmology in the next 5 years	Disagree/Neutral	18	47%	76	46%	48	45%	0.96
	Agree	20	53%	88	54%	59	55%	
AI can maintain the confidentiality of the patients’ data based on e-health data security & privacy governance protocols	Disagree/Neutral	7	18%	44	27%	26	24%	0.55
	Agree	31	82%	120	73%	81	76%	
AI will increase the accessibility of DR screening and follow-up at the patient’s own convenience	Disagree/Neutral	3	8%	25	15%	11	10%	0.31
	Agree	35	92%	139	85%	96	90%	
AI will increase detection of early stages DR and decrease the progression of advanced stages DR	Disagree/Neutral	5	13%	30	18%	11	10%	0.18
	Agree	33	87%	134	82%	96	90%	
AI will accelerate the management for patients requiring urgent intervention	Disagree/Neutral	8	21%	31	19%	11	10%	0.12
	Agree	30	79%	133	81%	96	90%	
AI will offer diabetic patients a safer environment for examination during and post COVID-19 pandemic	Disagree/Neutral	4	11%	29	18%	12	11%	0.25
	Agree	34	89%	135	82%	95	89%	
AI will further support non-essential contact between eye care providers and patients during and post COVID-19 pandemic	Disagree/Neutral	7	18%	25	15%	11	10%	0.36
	Agree	31	82%	139	85%	96	90%	

Values in bold denote statistical significance, which is represented by a *p*-value < 0.05.

The 14 Likert scale (ranging from 1 to 5) statements regarding the potential impact of AI application were categorized into three categories. The Likert Scale score of all the statements reflecting each category was summed up, and the mean score was compared among the demographic characteristics and clinical practice of the respondents (Tables 7, 8). Nine statements were included in the advantages of AI applications (max score = 45) (Table 7). It was found that respondents with more than 20 years of clinical experience had a higher score (37.5 ± 5.0) as compared to those with 5 to 20 years of experience (35.7 ± 4.9) (*p* = 0.008). The respondents who agreed with the statement that e-health apps had increased the efficiency of their clinical practice had a higher score (37.6 ± 4.4) as compared to those who were neutral/disagreed (33.7 ± 5.6) (*p* < 0.001). The respondents who rated their understanding of AI as above average/excellent had a higher score (37.4 ± 5.0) as compared to those who rated their knowledge as very poor/below average (35.4 ± 5.0) (*p* = 0.003). There were also significantly higher scores for those who agreed that AI and telemedicine could be used interchangeably (37.7 ± 4.6 vs. 34.6 ± 5.0) (*p* < 0.001) as well as for those who agreed that using e-health apps promoted their acceptance of utilizing AI (37.7 ± 4.3 vs. 32.8 ± 5.5) (*p* < 0.001).

**Table 7:** Impact of characteristics of perceived AI knowledge and clinical practice of study sample on perception of advantages of AI application in diabetic retinopathy screening.

		*n*	Advantages of AI application (max = 45)	Value of *p*
			Mean ± SD	
Current profession	Ophthalmologist	267	36.5 ± 4.9	0.67
	Other	42	36.8 ± 5.3	
Clinical experience in eye care services	<5 years	38	37.2 ± 4.6	**0.008**
	5–20 years	164	35.7 ± 4.9	
	>20 years	107	37.5 ± 5.0	
Gender	Male	206	36.2 ± 5.2	**0.097**
	Female	103	37.2 ± 4.4	
Using e-health apps in your clinical practice prior and during the COVID-19 pandemic	No	104	36.0 ± 4.9	0.22
	Yes	205	36.8 ± 5.0	
Have e-health apps increased the efficiency of your clinical practice?	Disagree/Neutral	43	33.7 ± 5.6	**<0.001**
	Agree	189	37.6 ± 4.4	
How would you rate your understanding of AI?	Very poor/Below average	35	35.4 ± 5.0	**0.003**
	Average	109	35.5 ± 4.7	
	Above average/Excellent	165	37.4 ± 5.0	
AI and telemedicine can be used interchangeably	Disagree/Neutral	115	34.6 ± 5.0	**<0.001**
	Agree	194	37.7 ± 4.6	
Using e-health apps promotes my understanding and acceptance of utilizing AI in healthcare	Disagree/Neutral	67	32.8 ± 5.5	**<0.001**
	Agree	228	37.7 ± 4.3	

Values in bold denote statistical significance, which is represented by a *p*-value < 0.05.

**Table 8:** Impact of characteristics of perceived AI knowledge and clinical practice of study sample on perception of AI clinical application effect on labor market.

		*n*	Effect on labor market (max = 10)	Value of *p*
			Mean ± SD	
Current profession	Ophthalmologist	267	5.2 ± 1.8	0.68
	Other	42	4.7 ± 1.6	
Clinical experience in eye care services	<5 years	38	4.9 ± 1.6	0.73
	5–20 years	164	5.1 ± 1.8	
	>20 years	107	5.1 ± 1.9	
Gender	Male	206	5.1 ± 1.9	0.59
	Female	103	5.0 ± 1.7	
Using e-health apps in your clinical practice prior and during the COVID-19 pandemic	No	104	5.2 ± 1.8	0.47
	Yes	205	5.0 ± 1.8	
Have e-health apps increased the efficiency of your clinical practice?	Disagree/Neutral	43	5.5 ± 1.9	**0.023**
	Agree	189	4.8 ± 1.7	
How would you rate your understanding of AI?	Very poor/Below average	35	5.2 ± 1.4	0.27
	Average	109	5.3 ± 1.6	
	Above average/Excellent	165	4.9 ± 2.0	
AI and telemedicine can be used interchangeably	Disagree/Neutral	115	5.8 ± 1.7	**<0.001**
	Agree	194	4.7 ± 1.8	
Using e-health apps promotes my understanding and acceptance of utilizing AI in healthcare	Disagree/Neutral	67	5.8 ± 1.6	**<0.001**
	Agree	228	4.8 ± 1.7	

Values in bold denote statistical significance, which is represented by a *p*-value < 0.05.

The implementation and education scores were determined by combining three statements (max = 15). Ophthalmologists had a lower score (10.4 ± 2.7) compared to those in other specialties (11.4 ± 2.2) (*p* = 0.03). The respondents who agreed that e-health apps increased the efficiency of their clinical practice had a higher score (11.0 ± 2.6) than those who disagreed (9.5 ± 2.0) (*p* < 0.001). The implementation scores were also higher for those who agreed that AI and telemedicine could be used interchangeably (11.1 ± 2.5 vs. 9.6 ± 2.6) (*p* < 0.001) as well as for those who agreed that using e-health apps promoted their acceptance of utilizing AI (10.8 ± 2.6 vs. 9.6 ± 2.4) (*p* < 0.001).

Table 8 compares of the mean scores for the effect on the labor market that was calculated by combining two statements (max score = 10). A significant difference was identified for those who disagreed that e-health apps increased the efficiency of their clinical practice (5.5 ± 1.9 vs. 4.8 ± 1.7) (*p* = 0.023). There were also significant differences for those who disagreed with the statement that AI and telemedicine could be used interchangeably (5.8 ± 1.6 vs. 4.7 ± 1.8) (*p* < 0.001) and for those who disagreed that using e-health apps promoted their understanding and acceptance of utilizing AI (5.8 ± 1.6 vs. 4.8 ± 1.7) (*p* < 0.001).

## 4 Discussion

This survey was conducted to understand the perceptions of ophthalmologists and primary care physicians, family physicians, and endocrinologists about AI-based DR screening, as these physicians are involved in managing diabetes and its complication of diabetic retinopathy. Furthermore, understanding the perception and knowledge of clinicians about this new-evolving technology is crucial for AI developers, professional bodies, and policymakers to implement such advancements into the health system. To our knowledge, this is the first survey of this scale to investigate clinicians’ perceptions of AI-based DR screening in Saudi Arabia.

### 4.1 Perceived knowledge and use of artificial intelligence in clinical practice

Our study revealed that 55% of ophthalmologists and other physicians in Saudi Arabia reported a good level of knowledge about AI-based DR screening, and 45% rated their level of knowledge of AI from average to very poor. Despite most participants reporting that they were knowledgeable about AI, our results suggest an apparent misconception of AI and telemedicine among respondents, as 63% agreed that AI and telemedicine could be used interchangeably. Perceived knowledge reported in our study was similar to another study conducted in Riyadh, Saudi Arabia, which explored the perceptions and knowledge of AI application in healthcare among 70 primary care physicians and found a moderate level of acceptance of AI application along with a lack of knowledge about AI by the participants (39). Another study conducted in Australia and New Zealand across three different specialties (ophthalmology, radiology, and dermatology) reported similar findings, as most clinicians rated their knowledge of AI as being average, with most respondents reporting not having used AI technology in the clinical setting before (40). In addition, radiologists and radiology residents in the US reported a lack of knowledge of AI as they were not up to date with the latest advancements in AI in radiology (41). It is likely that increasing physician workloads may impact intrinsic motivation to stay up to date on technological advances that are less applicable to current practice. Such an observation highlights the importance of integrating AI clinical advancements in the curricula of medical training.

Besides, when the clinical experience was considered, we saw varying trends in perceived knowledge among ophthalmologists. When asked to rate their understanding of AI, a significantly lower percentage (34%) of the <5 years of experience group rated themselves as above average/excellent as compared to those with 5–20 years (55%) and > 20 years (57%) of experience (*p* = 0.014), reflecting that most of the knowledge about AI in ophthalmology came from exposure rather than the academic curriculum. This observation is supported by the perception survey of ophthalmologists in the United States, as the average experience of responding ophthalmologists was 22 years of experience, and they rated their understanding of AI as high (89%). Furthermore, 75% indicated that there should be formal courses and teachings regarding AI technology during medical school and residency training (42). Moreover, 72% of ophthalmologists with >20 years of experience considered that AI and telemedicine could be used interchangeably compared to 60% in the 5–20 years and 47% in the group with <5 years of experience (*p* = 0.017). Such results reflect the lack of AI literacy even among the group with the highest reported knowledge of AI. These findings suggest that training involving AI should be more standardized among residency programs.

Our results also showed that ophthalmologists and other physicians reported similar perceived knowledge about the use of AI in screening for diabetic retinopathy. In addition, there was a similar belief about e-health apps being helpful in promoting the understanding and acceptance of introducing AI in clinical practice. Our findings suggest a great need to address the deficit in the perceived understanding of the definition, capacity, and functions of AI. This could be achieved by introducing AI learning and exposure at the level of medical school along with incorporating AI in clinical training and upgrading the courses of practicing physicians (43).

### 4.2 Perceived impact of artificial intelligence on the profession

The perceived impact of AI is inconsistent in the documented literature. An online questionnaire conducted by Oh et al. and distributed to 669 participants showed that doctors have positive attitudes toward AI implementation in the medical field. Most of the surveyed physicians assumed that AI would not replace their roles (44). Previous studies (41, 44–46) further proved these positive sentiments toward AI implementation across several medical specialties. Among most studies, there seems to be a consensus among clinicians that AI implementation would drastically improve the efficiency of screening by reducing administrative burden and increasing the volume of images graded (47, 48).

On the other hand, the futurists Brougham and Haar expect that smart technology algorithms (STARA), and robotics could replace nearly one-third of all existing jobs by 2025 (49). This worry also extends to clinicians, with some believing that the job market would be cannibalized by the increased adoption of AI (46). Other concerns regarding AI include legal and ethical considerations growing increasingly more problematic (40, 50). Such a problem is consistent with the technology’s impact on the needs of the future workforce (41, 48). However, other surveys indicated that introducing AI would have minimal impact on workforce needs over the next decade (51).

Our study showed an overall positive attitude toward AI from participating physicians. In general, participants of our survey perceived the advantages of using AI in DR with agreement as all the statements about the impact of AI application in DR screening received >75% agreement. The four statements with the highest agreement among respondents were, respectively:

1. *“AI will increase the accessibility of DR screening and follow-up at the patient’s convenience”* (88%). This finding is very similar to the results in the study conducted in Australia and New Zealand, which revealed that ophthalmologists and dermatologists reported improved access to disease screening was the most significant perceived advantage of AI (40).
2. *“AI will provide a safer environment for examination and supporting non-essential contact between eye care providers and patients during and after the COVID-19 pandemic”* (86%).
3. *“AI would increase detection of early stages of DR and decrease the progression of advanced stages”* (85%).
4. *“AI would accelerate the management of patients requiring urgent intervention”* (84%).

In contrast, in a study by Abdullah R, 65% of the surveyed Primary care physicians in Saudi Arabia believed that AI would not be able to produce high-quality clinical data (39). This also mimics the findings of another paper that reported concerns on the diagnostic reliability of AI-based screening (42). Interestingly, these subjective findings go against some of the more recent evidence discussed earlier which concluded that AI-based screening rivals and in some cases is more efficacious than human graders (23). One theory for the discrepancy between our study and the aforementioned could be attributed to the COVID-19 pandemic, which has accelerated the public’s acceptance of technological and AI-based healthcare. In addition, it may be reasonable to consider that as time progresses, physicians tend to become more knowledgeable about common technological advancements, theoretically meaning that self-reported knowledge would increase when comparing older studies to more recent ones.

Analysis of our results revealed the perception of the impact of AI in DR screening is significantly related to the perceived level of AI knowledge, as those who rated their knowledge of AI as above average/excellent had a higher tendency to perceive the potential impact positively compared to those who rated their understanding as very poor/below average (*p* = 0.003). Among ophthalmologists, however, experience played a role in the acceptance of the advantages as it was found that respondents with more than 20 years of clinical experience had a higher score (37.5 ± 5.0) as compared to those with 5 to 20 years of experience (35.7 ± 4.9) (*p* = 0.008). To our knowledge, this is the first study to report the correlation between the level of knowledge of AI-based DR screening and the level of positivity toward AI implementation and advantages, also suggesting increased familiarity with this new horizon of medicine is likely to improve confidence in applying widespread screening programs involving AI technology.

Our analysis did reveal, however, a subset of respondents who felt that the implementation of AI would negatively influence the job market. 78% of e-health app users in clinical practice agreed that AI will be a competitor to diabetic clinical practitioners compared to 63% of non-users (*p* = 0.004). This effect was most apparent among those who believed that e-health apps increased the efficiency of their clinical practice (*p* = 0.023) and those who disagreed that using e-health apps promoted their understanding and acceptance of utilizing AI (*p* < 0.001).

### 4.3 Use of e-health apps

The correlation of using e-health apps during clinical practice was investigated heavily and showed significance. Users of e-health apps agreed with the statements that AI will spare specialists’ efforts spent in triaging, and that AI frees up specialists’ time to better utilize their surgical skills effectively compared to non-users (*p* = 0.002, *p* < 0.001, respectively). Those who agreed that using e-health apps promoted their acceptance of utilizing AI had a higher perception that AI would be advantageous (*p* < 0.001). We found that no previous studies in the literature investigated the prior use of technology as a factor that determines the acceptance of AI-based DR screening advantages.

In addition, experience played a role in accepting technology in clinical practice. Ophthalmology residents with less than five years of experience showed the highest tendency to accept e-health apps in clinical practice, as 96% of them agreed that e-health apps increased the efficiency in their clinical practice as compared to 78% in those with 5–20 years of experience and 88% in those with over 20 years of experience (*p* = 0.04). Itersum et al. investigated technology acceptance through the development of a qualitative model. Several factors were found to affect technology acceptance; those include individual user preferences and the characteristics of the technology organization used at the workplace.

### 4.4 Concerns about the use of artificial intelligence

Physicians who participated in the survey agreed the least with the statement regarding AI maintaining the confidentiality of the patients’ data based on e-health data security & privacy governance protocols (75%). This finding parallels another study conducted in Australia and New Zealand, which revealed that physicians were concerned about medical liability incurred from machine errors (40).

Concerns were also raised about the trust in data security and the impacts of AI on healthcare job security. Ethics surrounding the use of AI in healthcare have been debatable (52), perhaps due to the challenges in ensuring data privacy and proper data utilization, specifically during data collection modes conducted through third-party apps (53). Moreover, regardless of the privacy and security measures, the increasing reports of data leaks and vulnerabilities in electronic medical record databases erode people’s trust. Future security and transparency policies and measures could consider the use of blockchain technology, and privacy laws should be adequately delineated and transparent (54).

### 4.5 Preparedness for the introduction of artificial intelligence into clinical practice

Our findings showed that physicians in Saudi Arabia were hesitant to believe that AI would be applied in clinical practice within the next five years, as there was just over 50% agreement with the statements regarding their organizations’ willingness to educate healthcare workers in the use of AI and educating the public. Moreover, only 48% agreed that their organizations would invest in AI in the next five years. In another survey, 68% of pediatric ophthalmologists in the United States reported their interest in adopting AI in their clinical practice (42), which may reflect greater awareness of healthcare trends in the West. Furthermore, the use of e-health apps promoted the belief that AI could be implemented in the next five years. Those who agreed that e-health apps increased the efficiency of their clinical practice and promoted their acceptance of utilizing AI showed more acceptance of the idea (*p* < 0.001).

### 4.6 Perceptions of ophthalmologists vs. other physicians

Our study did not find any statistically significant differences between the responses of ophthalmologists and other physicians (primary care physicians, family physicians, and endocrinologists) regarding perceived knowledge and use of artificial intelligence in clinical practice, use of e-health apps, concerns about the use of artificial intelligence, and preparedness for the introduction of artificial intelligence into clinical practice. When comparing the perceived impact of artificial intelligence among different professions, a statistically greater proportion of non-ophthalmologists agreed with two of the thirteen statements: that their organization would be likely to train healthcare workers, and that the organization would likely educate the public about the use of AI in the next five years. However, this difference is likely attributed to the small sample size of non-ophthalmologist respondents in our study.

### 4.7 Strengths and limitations

#### 4.7.1 Strengths

This study is the first in Saudi Arabia to assess the perspective of ophthalmologists and other physicians involved in taking care of DR patients about AI screening programs. Our study included several factors which could influence the views of physicians. Understanding such perceptions is fundamental for successfully implementing AI in clinical practice. Considering the outcomes of this study will help policymakers and healthcare developers in Saudi Arabia plan strategies to educate and train physicians in the future to meet their expectations.

#### 4.7.2 Limitations

There were several limitations to be considered. As responses to questions about self-assessment of knowledge and definitions were collected from participants voluntarily, recall bias and misunderstanding would be expected to affect the results. Thus, these responses may not represent the views of physicians in Saudi Arabia in general, but rather the study sample perspectives. Another point to consider is the applicability of our findings to other countries, owing to the possible differences in knowledge among different study populations. There is also the possibility that non-ophthalmologists were underrepresented by our analysis owing to their disproportionate number compared to ophthalmologist respondents. Finally, the survey design does not include open-ended questions, and response options are limited to multiple choices. Therefore, the results of this study may not constitute a comprehensive perspective of participants.

### 4.8 Conclusion

This study aimed to assess the perception of ophthalmologists and physicians involved in diabetic eye care on the knowledge, advantages, and concerns of AI application in diabetic retinopathy screening, as this technology has not been implemented in clinical practice yet in Saudi Arabia. It is very crucial to understand the perception and expectations of stakeholders for successful strategic planning and application.

Physicians with more experience regarded their AI-related knowledge to be higher than their less experienced peers. Moreover, the use of e-health apps showed a significant effect on the perceived knowledge of AI in clinical practice, as those with more knowledge and clinical experience had a higher acceptance rate and belief in better clinical efficiency. Interestingly, despite the tendency for participants to rate themselves highly in terms of knowledge, many participants mistakenly agreed that telemedicine was interchangeable with AI, which possibly reflects that even among the subset of respondents who rate their knowledge the highest, there remains a degree of misinformation present.

In addition, ophthalmologists and other physicians generally agreed with the benefits of AI in diabetic retinopathy screening, believing it would increase healthcare efficiency. However, there were concerns about decreasing the workforce of physicians after AI implementation and whether this technology can maintain the confidentiality of clinical data. Physicians showed a high agreement regarding AI being a safe solution for clinical practice, especially during times of a pandemic such as COVID-19, as it provides a safer environment for patients and providers, as well as minimizes disruptions to screening timetables.

Our findings suggest a great need to address the deficits in the physicians’ insights regarding the knowledge and functions of AI. This could be achieved by introducing AI learning and exposure at the level of medical school, incorporating AI in clinical training, and hosting talks by field pioneers at national conferences. Technological literacy seems to significantly (and positively) influence the ability of clinicians to “warm up” to the idea of working alongside AI in a clinical setting, as evidenced by e-health app users’ increased tendency to agree more with positive statements about the use of AI. The authors believe that a larger institutional push for the use of e-health apps would likely accelerate a change in the mentality of physicians in a myriad of medical fields.

Our findings also demonstrated that AI is valued for its potential to accelerate diagnosis, waiting time, referrals, and several other routine tasks to run more efficiently in clinical practice, which allows clinicians to use their time and resources to prioritize more complex tasks, such as surgical operations and personally interacting with patients. This reduces the risk of exhaustion, job dissatisfaction, and shortage of manpower. This becomes of prime importance, considering that ARIAS has reached a diagnostic accuracy level that is on par with human graders, while simultaneously being more time efficient. This is especially important in areas with limited access to screening programs and preventative eye care.

Our findings also corroborate some of the concerns in other studies regarding AI use. Information privacy is a growing concern worldwide, and global distrust of AI is a matter that must be addressed before the widespread use of AI in clinical practice in the field of ophthalmology. This could be achieved by addressing the medical community’s concerns of information privacy and managing their expectations, while slowly increasing technological literacy.

Overall, addressing the perception of ophthalmologists toward AI-based DR, while highlighting its potential contribution to patients’ care could have a positive impact on patients’ satisfaction, efficient delivery of health care and overall disease outcome.

### 4.9 Suggestions for the future

The technological advances that the COVID-19 pandemic has accelerated, as well as the advent of generative AI technology such as ChatGPT, have created a major paradigm shift. Both academic circles, as well as the public eye, have become all the more attentive to the capabilities of artificial intelligence. We expect that awareness of AI will only increase in the future. Major issues like data security and privacy will continue to remain a large hurdle and will likely be a major limiting factor in the large-scale and realistic applicability of AI in clinical practice. Large leaps in data security must coincide with the growing computational prowess of AI in order to allow us to safely integrate it into daily clinical workflows. We highly encourage more research to be performed in this domain in the future. Our study has demonstrated that increasing technological literacy (in the form of e-health app use) increases the likelihood that providers will view AI implementation as a net positive. Reflecting on these results, the authors believe that the next logical step in furthering our understanding of the interplay between physician perception and the implementation of AI is through studying the controlled application of artificial intelligence. Cross-sectional surveys are inherently limited by the possible homogeneity of the studied population. In addition, individual perceptions of artificial intelligence can be informed by many factors including exposed media, life experience, and academic background. Future prospective studies on the diagnostic accuracy of artificial intelligence may be further modified by implementing pre-study and post-study perception surveys to be completed by the involved healthcare providers. This would allow us to understand how AI implementation modulates the perceptions of a controlled group of providers and allow us to more carefully simulate the response healthcare providers will have to real-world implementation of this technology. One avenue that stakeholders can implement to accelerate provider awareness of the utility of AI is to encourage the use of e-health apps and smartphone-based decision-making tools more widely. The mounting evidence on the utility of AI seems to point toward a generational leap in healthcare efficiency. This emphasizes even more the need to be measured in the research that is produced in this domain, and to carefully dissect potential barriers to implementation.

## Data availability statement

The raw data supporting the conclusions of this article will be made available by the authors, without undue reservation.

## Ethics statement

The studies involving humans were approved by the Alfaisal University College of Medicine Institutional Review Board. The studies were conducted in accordance with the local legislation and institutional requirements. The participants provided their written informed consent to participate in this study.

## Author contributions

AB: Conceptualization, Investigation, Methodology, Project administration, Writing – original draft, Writing – review & editing, Validation. OM: Writing – original draft, Writing – review & editing. HJ: Writing – original draft, Writing – review & editing. MA: Writing – original draft. KH: Conceptualization, Investigation, Methodology, Writing – original draft. AO: Conceptualization, Investigation, Methodology, Project administration, Supervision, Writing – review & editing. SA-H: Conceptualization, Investigation, Methodology, Project administration, Supervision, Writing – review & editing.

## Footnotes

^1^IDx-Dr, Google neural network, respectively.

## Supplementary material

### SUPPLEMENTARY FIGURE S1

Principal Components Analysis (PCA).

### SUPPLEMENTARY FIGURE S2

Summary of research pipeline.
