# Supplementary material for: The application of artificial intelligence in diabetic retinopathy screening: a Saudi Arabian perspective
Source: Front Med (Lausanne). 2023 Nov 22;10:1303300. doi: 10.3389/fmed.2023.1303300 (PMC10703427; doi:10.3389/fmed.2023.1303300)
Supplement: Supplementary file 1 [file Data_Sheet_1.docx]

**Appendix**

**Appendix 1: Questionnaire of the research**

1. ***Consent***

This survey is distributed to healthcare professionals involved in eye care. The outcomes of this study are essential to understand the perception and acceptance of eye care practitioners of AI application in diabetic retinopathy screening, and guidance of clinical policies and procedures for future implementation in clinical settings.

Unless otherwise specified, the term artificial intelligence (AI) is used to describe the use of computer systems to perform tasks normally requiring human expertise. Applications of AI in healthcare include examples such as image analysis for disease detection, triage, and language-recognition for diagnosis.

Your participation is voluntary. Survey respondents will remain anonymous. Your responses will be kept confidential and used for research purposes only. Answering this survey is your agreement to participate.

1. ***Study Questionnaire***

*** Required**

1. Current profession *

*Mark only one oval.*

o Ophthalmologist (inclusive of training)

o Other (e.g. Endocrinologists, Primary Care Physicians): Please specify ……………..

2. Clinical experience in eye care services *

*Mark only one oval.*

o <5 years (inclusive of training) 5-20 years

o More than 20 years

3. Practicing in Saudi Arabia? *

*Mark only one oval.*

o Yes

o No

4. Gender *

*Mark only one oval.*

o Male

o Female

5. Have you been using e-health apps in your clinical practice prior and during the COVID-19 pandemic? *

*Mark only one oval.*

o Yes

o No

6.Have e-health apps increased the efficiency of your clinical practice? *

(if your answer to previous question is “No”, please choose “N/A”)

*Mark only one oval.*

o Strongly agree

o Agree

o Neutral

o Disagree

o Strongly disagree

o N/A

7. How would you rate your understanding of AI? *

*Mark only one oval.*

o Excellent

o Above average

o Average

o Below average

o Very poor

8. AI and telemedicine can be used interchangeably *

*Mark only one oval.*

o Strongly agree

o Agree

o Neutral

o Disagree

o Strongly disagree

9. Using e-health apps promotes my understanding and acceptance of utilizing AI in healthcare *

*Mark only one oval.*

o Strongly agree

o Agree

o Neutral

o Disagree

o Strongly disagree

o N/A

10. AI will be a competitor to diabetic clinical practitioners *

*Mark only one oval.*

o Strongly agree

o Agree

o Neutral

o Disagree

o Strongly disagree

11. AI will decrease the number of the workforce needed for diabetic clinical eye care *

*Mark only one oval.*

o Strongly agree

o Agree

o Neutral

o Disagree

o Strongly disagree

12. AI will complement my organization's diabetic clinical eye practice *

*Mark only one oval.*

o Strongly agree

o Agree

o Neutral

o Disagree

o Strongly disagree

13. AI will spare specialists' efforts spent in triaging DR to better utilize time for more surgical procedures *

*Mark only one oval.*

o Strongly agree

o Agree

o Neutral

o Disagree

o Strongly disagree

14. AI will increase the encounters for specialists to better utilize their surgical skills efficiently *

*Mark only one oval.*

o Strongly agree

o Agree

o Neutral

o Disagree

o Strongly disagree

15. My organization is likely to invest in AI in the clinical practice for DR in the next 5 years *

*Mark only one oval.*

o Strongly agree

o Agree

o Neutral

o Disagree

o Strongly disagree

16. My organization is likely to train healthcare workers in the use of AI in the next 5 years *

*Mark only one oval.*

o Strongly agree

o Agree

o Neutral

o Disagree

o Strongly disagree

17. My organization is likely to educate the public regarding the use of AI in Ophthalmology in the next 5 years *

*Mark only one oval.*

o Strongly agree

o Agree

o Neutral

o Disagree

o Strongly disagree

18. AI can maintain the confidentiality of the patients' data based on e-health data security & privacy governance protocols *

*Mark only one oval.*

o Strongly agree

o Agree

o Neutral

o Disagree

o Strongly disagree

19. AI will increase the accessibility of DR screening and follow-up at the patient's own convenience *

*Mark only one oval.*

o Strongly agree

o Agree

o Neutral

o Disagree

o Strongly disagree

20. AI will increase detection of early stages DR and decrease the progression of advanced stages DR *

*Mark only one oval.*

o Strongly agree

o Agree

o Neutral

o Disagree

o Strongly disagree

21. AI will accelerate the management for patients requiring urgent intervention *

*Mark only one oval.*

o Strongly agree

o Agree

o Neutral

o Disagree

o Strongly disagree

22. AI will offer diabetic patients a safer environment for examination during and post COVID-19 pandemic *

*Mark only one oval.*

o Strongly agree

o Agree

o Neutral

o Disagree

o Strongly disagree

23. AI will further support non-essential contact between eye care providers and patients during and post COVID-19 pandemic *

*Mark only one oval.*

o Strongly agree

o Agree

o Neutral

o Disagree

o Strongly disagree

**Appendix 2: Reliability study of the questionnaire**

Scale: All Q10-23

| **Case Processing Summary** | | | |
| --- | --- | --- | --- |
|  | | N | % |
| Cases | Valid | 320 | 100.0 |
|  | Excluded^a^ | 0 | 0.0 |
|  | Total | 320 | 100.0 |
| a. Listwise deletion based on all variables in the procedure. | | | |

| **Reliability Statistics** | |
| --- | --- |
| Cronbach's Alpha | N of Items |
| 0.887 | 14 |

| **Item-Total Statistics** | | | | |
| --- | --- | --- | --- | --- |
|  | Scale Mean if Item Deleted | Scale Variance if Item Deleted | Corrected Item-Total Correlation | Cronbach's Alpha if Item Deleted |
| 10. AI will be a competitor to diabetic clinical practitioners | 50.83 | 49.211 | 0.329 | 0.894 |
| 11. AI will decrease the number of the workforce needed for diabetic clinical eye care | 50.43 | 48.076 | 0.457 | 0.886 |
| 12. AI will compliment my organization's diabetic clinical eye practice | 50.09 | 47.797 | 0.674 | 0.875 |
| 13. AI will spare specialists' efforts spent in triaging DR to better utilize time for more surgical procedures | 50.08 | 47.405 | 0.642 | 0.876 |
| 14. AI will increase the encounters for specialists to better utilize their surgical skills efficiently | 50.14 | 48.849 | 0.571 | 0.879 |
| 15. My organization is likely to invest in AI in the clinical practice for DR in the next 5 years | 50.63 | 46.774 | 0.548 | 0.881 |
| 16. My organization is likely to train healthcare workers in the use of AI in the next 5 years | 50.53 | 46.990 | 0.590 | 0.878 |
| 17. My organization is likely to educate the public regarding the use of AI in Ophthalmology in the next 5 years | 50.53 | 47.830 | 0.546 | 0.880 |
| 18. AI can maintain the confidentiality of the patients' data based on e-health data security & privacy governance protocols | 50.15 | 49.219 | 0.567 | 0.880 |
| 19. AI will increase the accessibility of DR screening and follow-up at the patient's own convenience | 49.93 | 48.700 | 0.678 | 0.876 |
| 20. AI will increase detection of early stages DR and decrease the progression of advanced stages DR | 49.91 | 48.246 | 0.662 | 0.876 |
| 21. AI will accelerate the management for patients requiring urgent intervention | 49.92 | 47.962 | 0.644 | 0.876 |
| 22. AI will offer diabetic patients a safer environment for examination during and post COVID-19 pandemic | 49.92 | 48.604 | 0.610 | 0.878 |
| 23. AI will further support non-essential contact between eye care providers and patients during and post COVID-19 pandemic | 49.89 | 48.428 | 0.614 | 0.878 |

| **Appendix 3: Principle component analysis** |
| --- |
|  |
|  |
| **Scale: Advantages of AI application to clinical practice** |

|  |  |  |  |
| --- | --- | --- | --- |

| **Case Processing Summary** | | | |
| --- | --- | --- | --- |
|  | | N | % |
| Cases | Valid | 320 | 100.0 |
|  | Excluded^a^ | 0 | 0.0 |
|  | Total | 320 | 100.0 |
| a. Listwise deletion based on all variables in the procedure. | | | |

| **m** | |
| --- | --- |
| Cronbach's Alpha | N of Items |
| 0.816 | 4 |

| **Item-Total Statistics** | | | | |
| --- | --- | --- | --- | --- |
|  | Scale Mean if Item Deleted | Scale Variance if Item Deleted | Corrected Item-Total Correlation | Cronbach's Alpha if Item Deleted |
| 12. AI will compliment my organization's diabetic clinical eye practice | 12.09 | 3.750 | 0.626 | 0.773 |
| 13. AI will spare specialists' efforts spent in triaging DR to better utilize time for more surgical procedures | 12.07 | 3.396 | 0.680 | 0.747 |
| 14. AI will increase the encounters for specialists to better utilize their surgical skills efficiently | 12.14 | 3.805 | 0.609 | 0.781 |
| 21. AI will accelerate the management for patients requiring urgent intervention | 11.92 | 3.701 | 0.630 | 0.771 |

**Scale: Implementation & education of AI screening in next 5 years**

| **Case Processing Summary** | | | |
| --- | --- | --- | --- |
|  | | N | % |
| Cases | Valid | 320 | 100.0 |
|  | Excluded^a^ | 0 | 0.0 |
|  | Total | 320 | 100.0 |
| a. Listwise deletion based on all variables in the procedure. | | | |

| **Reliability Statistics** | |
| --- | --- |
| Cronbach's Alpha | N of Items |
| 0.918 | 3 |

| **Item-Total Statistics** | | | | |
| --- | --- | --- | --- | --- |
|  | Scale Mean if Item Deleted | Scale Variance if Item Deleted | Corrected Item-Total Correlation | Cronbach's Alpha if Item Deleted |
| 15. My organization is likely to invest in AI in the clinical practice for DR in the next 5 years | 7.09 | 3.064 | 0.797 | 0.918 |
| 16. My organization is likely to train healthcare workers in the use of AI in the next 5 years | 6.99 | 3.138 | 0.881 | 0.844 |
| 17. My organization is likely to educate the public regarding the use of AI in Ophthalmology in the next 5 years | 7.00 | 3.376 | 0.834 | 0.885 |

**Scale: Advantages of AI screening**

| **Case Processing Summary** | | | |
| --- | --- | --- | --- |
|  | | N | % |
| Cases | Valid | 320 | 100.0 |
|  | Excludeda | 0 | 0.0 |
|  | Total | 320 | 100.0 |
| a. Listwise deletion based on all variables in the procedure. | | | |

| **Reliability Statistics** | |
| --- | --- |
| Cronbach's Alpha | N of Items |
| 0.773 | 3 |

| **Item-Total Statistics** | | | | |
| --- | --- | --- | --- | --- |
|  | Scale Mean if Item Deleted | Scale Variance if Item Deleted | Corrected Item-Total Correlation | Cronbach's Alpha if Item Deleted |
| 18. AI can maintain the confidentiality of the patients' data based on e-health data security & privacy governance protocols | 8.31 | 1.538 | 0.540 | 0.768 |
| 19. AI will increase the accessibility of DR screening and follow-up at the patient's own convenience | 8.09 | 1.487 | 0.667 | 0.632 |
| 20. AI will increase detection of early stages DR and decrease the progression of advanced stages DR | 8.07 | 1.415 | 0.621 | 0.678 |

**Scale: Safeties of AI in COVID-19 pandemic**

| **Case Processing Summary** | | | |
| --- | --- | --- | --- |
|  | | N | % |
| Cases | Valid | 320 | 100.0 |
|  | Excluded^a^ | 0 | 0.0 |
|  | Total | 320 | 100.0 |
| a. Listwise deletion based on all variables in the procedure. | | | |

| **Reliability Statistics** | |
| --- | --- |
| Cronbach's Alpha | N of Items |
| 0.790 | 2 |

| **Item-Total Statistics** | | | | |
| --- | --- | --- | --- | --- |
|  | Scale Mean if Item Deleted | Scale Variance if Item Deleted | Corrected Item-Total Correlation | Cronbach's Alpha if Item Deleted |
| 22. AI will offer diabetic patients a safer environment for examination during and post COVID-19 pandemic | 4.18 | 0.556 | 0.653 |  |
| 23. AI will further support non-essential contact between eye care providers and patients during and post COVID-19 pandemic | 4.16 | 0.536 | 0.653 |  |

**Scale: The effect of AI on labor market**

| **Case Processing Summary** | | | |
| --- | --- | --- | --- |
|  | | N | % |
| Cases | Valid | 320 | 100.0 |
|  | Excluded^a^ | 0 | 0.0 |
|  | Total | 320 | 100.0 |
| a. Listwise deletion based on all variables in the procedure. | | | |

| **Reliability Statistics** | |
| --- | --- |
| Cronbach's Alpha | N of Items |
| 0.662 | 2 |

| **Item-Total Statistics** | | | | |
| --- | --- | --- | --- | --- |
|  | Scale Mean if Item Deleted | Scale Variance if Item Deleted | Corrected Item-Total Correlation | Cronbach's Alpha if Item Deleted |
| 10. AI will be a competitor to diabetic clinical practitioners | 2.35 | 0.988 | 0.497 |  |
| 11. AI will decrease the number of the workforce needed for diabetic clinical eye care | 2.76 | 1.168 | 0.497 |  |

**Appendix 4: Summary of the study sample’s central tendency**

Statistics

| Advantages of AI application (max=45) | | | Implementation and education of AI screening program in the next 5 years  (max=15) | Effect on labor market (max=10) |
| --- | --- | --- | --- | --- |
| N | Valid | 320 | 320 | 320 |
|  | Missing | 0 | 0 | 0 |
| Mean | | 36.65 | 10.54 | 6.89 |
| Median | | 36.00 | 11.00 | 7.00 |
| Std. Deviation | | 5.009 | 2.627 | 1.795 |
| Skewness | | -.367 | -.278 | -.390 |
| Std. Error of Skewness | | .136 | .136 | .136 |
| Kurtosis | | .346 | -.060 | -.209 |
| Std. Error of Kurtosis | | .272 | .272 | .272 |
| Minimum | | 21 | 3 | 2 |
| Maximum | | 45 | 15 | 10 |
