# Supplementary figures and images for: The application of artificial intelligence in diabetic retinopathy screening: a Saudi Arabian perspective
Source: Front Med (Lausanne). 2023 Nov 22;10:1303300. doi: 10.3389/fmed.2023.1303300 (PMC10703427; doi:10.3389/fmed.2023.1303300)

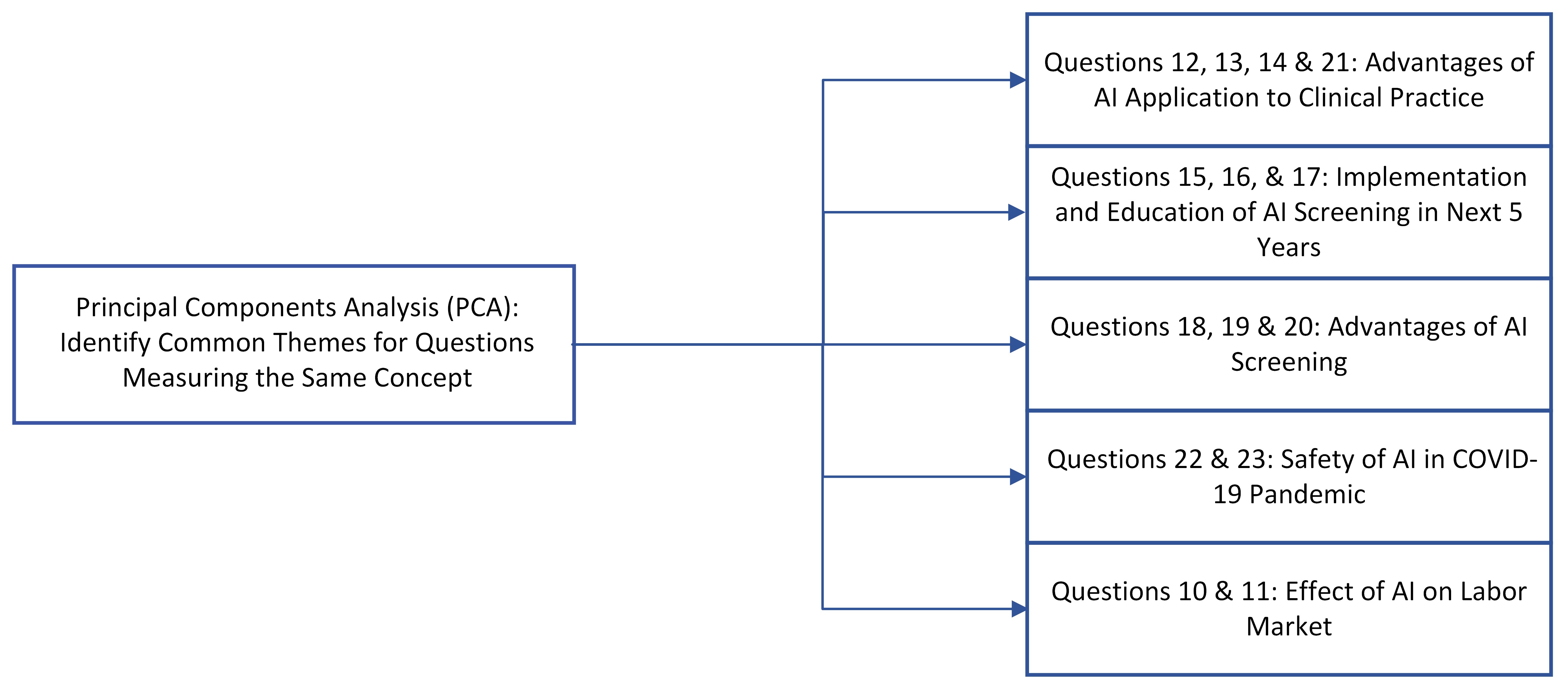

Supplement: Supplementary file 2 [file Image_1.jpeg]

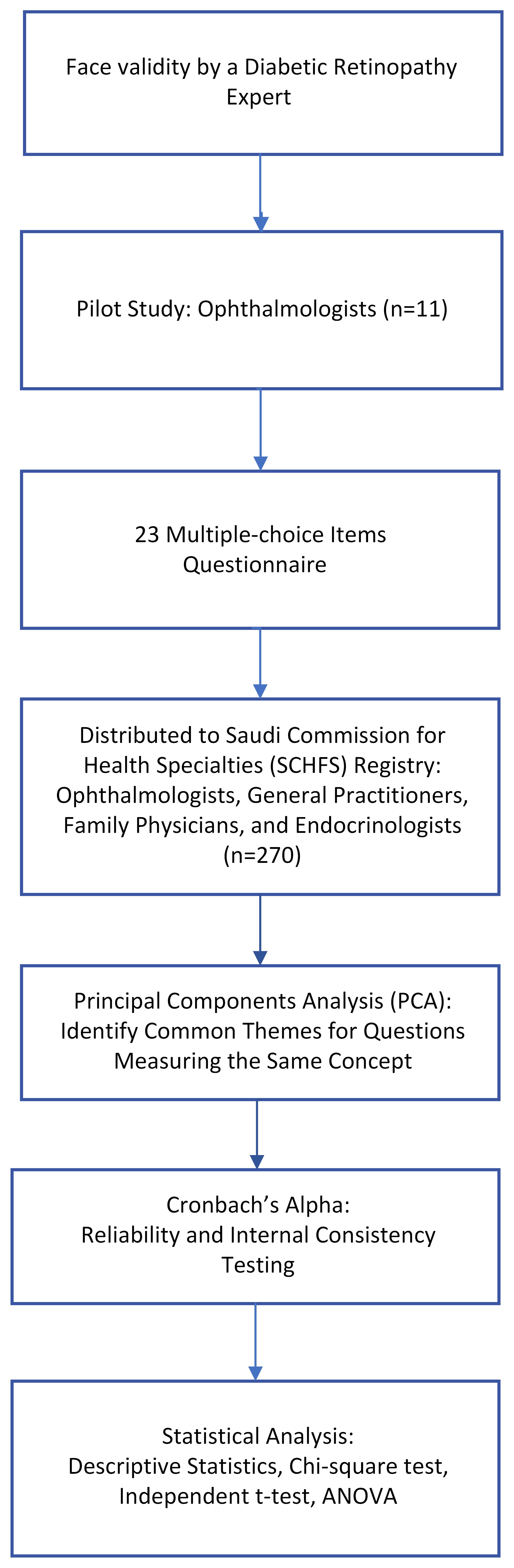

Supplement: Supplementary file 3 [file Image_2.jpeg]
